# Molecular Characterization of the Multiple Interactions of SpsD, a Surface Protein from *Staphylococcus pseudintermedius,* with Host Extracellular Matrix Proteins

**DOI:** 10.1371/journal.pone.0066901

**Published:** 2013-06-21

**Authors:** Giampiero Pietrocola, Joan A. Geoghegan, Simonetta Rindi, Antonella Di Poto, Antonino Missineo, Valerio Consalvi, Timothy J. Foster, Pietro Speziale

**Affiliations:** 1 Department of Molecular Medicine, Institute of Biochemistry, Pavia, Italy; 2 Department Microbiology, Moyne Institute of Preventive Medicine, Trinity College, Dublin, Ireland; 3 Department of Biochemical Sciences “A. Rossi Fanelli”, University of Rome “La Sapienza”, Roma, Italy; University of Kentucky College of Medicine, United States of America

## Abstract

*Staphylococcus pseudintermedius*, a commensal and pathogen of dogs and occasionally of humans, expresses surface proteins potentially involved in host colonization and pathogenesis. Here, we describe the cloning and characterization of SpsD, a surface protein of *S. pseudintermedius* reported as interacting with extracellular matrix proteins and corneocytes. A ligand screen and Western immunoblotting revealed that the N-terminal A domain of SpsD bound fibrinogen, fibronectin, elastin and cytokeratin 10. SpsD also interfered with thrombin-induced fibrinogen coagulation and blocked ADP-induced platelet aggregation. The binding site for SpsD was mapped to residues 395–411 in the fibrinogen γ-chain, while binding sites in fibronectin were localized to the N- and C-terminal regions. SpsD also bound to glycine- and serine-rich omega loops within the C-terminal tail region of cytokeratin 10. Ligand binding studies using SpsD variants lacking the C-terminal segment or containing an amino-acid substitution in the putative ligand binding site provided insights into interaction mechanism of SpsD with the different ligands. Together these data demonstrate the multi-ligand binding properties of SpsD and illustrate some interesting differences in the variety of ligands bound by SpsD and related proteins from *S. aureus*.

## Introduction

*Staphylococcus pseudintermedius* is a commensal and opportunistic pathogen of companion animals, especially dogs [1], [2], mainly causing skin infections such as pyoderma as well as surgical wound infections, urinary tract infections and otitis externa. Cases of infections in humans have occasionally been reported [3]–[6]. Methicillin-resistant *S. pseudintermedius* occurs widely [7], [8]. The complete genome sequences of two isolates of *S. pseudintermedius* are available [9], [10]. The strains are predicted to encode many putative virulence factors including toxins, extracellular enzymes such as lipases and proteases and surface proteins designated *S. pseudintermedius* surface proteins A-R (SpsA-R) [11] some of which are known to promote adhesion of the bacterium to desquamated skin epithelial cells (corneocytes) [12]–[14] and to components of the extracellular matrix [11], [15].

One such surface protein that is likely to be important in skin colonization and virulence is SpsD. The presence of SpsD on the bacterial cell surface promotes adhesion to fibrinogen (Fbg), fibronectin (Fn) and cytokeratin 10 (K10). Immunoglobulin G specific for SpsD occurs in dogs with pyoderma indicating that the protein is expressed during infection [11]. SpsD has many features that are typical of staphylococcal surface proteins called microbial surface components recognizing adhesive matrix molecules (MSCRAMMs) that are related to clumping factors (Clf) and fibronectin binding proteins (FnBPs) of *S. aureus* [11].

**Figure 1:**
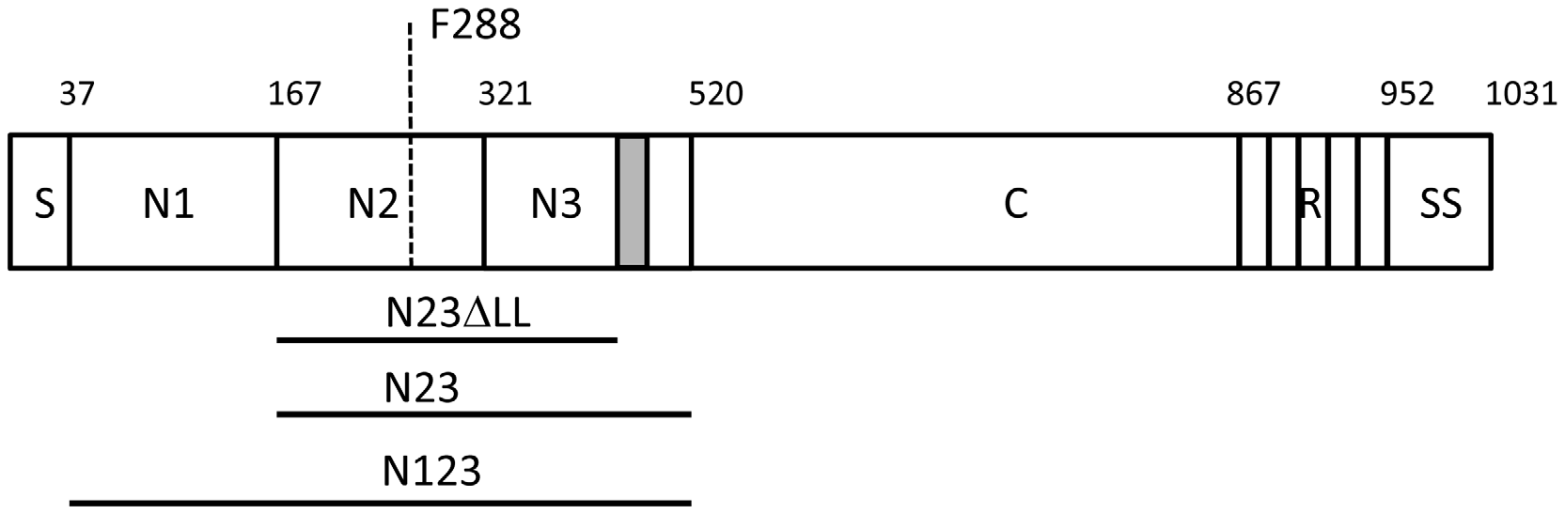
Schematic diagram of SpsD. The A domain modelled on the structure of ClfA spans residues 37–519 following the secretory signal sequence S. This is followed by a connecting region C (residues 521–867) and a repeat region R in strain ED99. The number of repeats varies from strain to strain resulting in proteins of slightly different sizes. A sorting signal (SS: LPXTG motif, hydrophobic domain and positively charged residues) occurs at the extreme C-terminus. The minimum ligand binding domain construct N2N3 spans residues 167–519. Residues comprising the ‘lock’ and ‘latch’ (grey box residues 490–499) are indicated (N23ΔLL). Residue F288 that is predicted to lie within the ligand binding trench is indicated by the dashed line.

The primary translation product of SpsD has an N-terminal secretory signal sequence and a C-terminal sorting signal comprising an LPXTG motif for sortase A-mediated linkage to peptidoglycan, a hydrophobic membrane-spanning domain and positively charged residues. The N-terminus of the mature protein comprises an A domain, part of which has sequence similarity to the A domains of ClfA and FnBPs (Figure 1).

The A domains of FnBPA and FnBPB show significant sequence similarity to ClfA and are predicted to fold into three independent subdomains, N1, N2 and N3. The ligand binding sites within ClfA, FnBPA and FnBPB are confined to the N2 and N3 subdomains and they each bind to the C-terminus of the γ-chain of Fbg most likely by the same “dock, lock and latch” mechanism [16]–[18]. X-ray crystal structure analysis of ClfA revealed that the apo form of the protein adopts an open conformation with an exposed hydrophobic trench located between separately folded N2 and N3 subdomains. Binding of the Fbg γ-chain peptide is initiated by docking of the ligand peptide in the trench. A flexible extension of the N3 subdomain redirects towards N2, covers the trench-bound peptide and locks it in place by β-strand complementation with a β sheet in subdomain N2. Structural and mechanistic studies strongly suggest that the N2N3 subdomains of FnBPA and FnBPB bind to the Fbg γ chain by the same mechanism. However differences exist in the repertoire of ligands bound in addition to Fbg, with ClfA recognizing complement factor I from human serum [19], [20], FnBPA and FnBPB both additionally binding to elastin [17], [21], [22] and subdomains N2N3 of FnBPB also binding to fibronectin [23].

Clumping factor B is another MSCRAMM of *S. aureus* with an A domain that is similar in structure and function to ClfA but which binds different ligands to ClfA and FnBPs by the dock, lock and latch mechanism [24]. ClfB binds to the glycine and serine-rich omega loops that occur in the C-terminal tail of cytokeratin 10 and throughout the corneocyte envelope protein loricrin [25], [26]. It also binds to a related sequence in the αC region of the α chain of Fbg [24], [27].

Located distally to the A domains of FnBPA and FnBPB is an extended unfolded region comprising 11 or 10 tandemly repeated domains, respectively, that bind to the N-terminal type I modules of Fn by the tandem β-zipper mechanism [28], [29]. In ClfA and ClfB this region is occupied by multiple repeats of the dipeptide Ser-Asp which have no known ligand binding function [30].

SpsD has been reported to promote bacterial adhesion to Fbg, K10 and Fn. In this study we set out to dissect SpsD and to localize and characterize its ligand binding region(s). We identified a region that is most closely related to the A domain of FnBPB of *S. aureus* that bound to these ligands and we provide insights into the ligand binding mechanism.

## Materials and Methods

### Bacterial Strains and Growth Conditions

*Escherichia coli* strain TOPP3 (Stratagene, La Jolla, CA, USA) was used as host for expression of recombinant proteins. *E. coli* strain TOPP3 was grown in Luria broth (LB) containing ampicillin (100 µg/ml). *S. pseudintermedius* strain 326 was isolated from canine pyoderma [15]. Staphylococcal cells were grown for the indicated times at 37°C in BHI (Brain Heart Infusion) broth (BD, Sparks, MD, USA) with shaking.

### Proteins

Fibronectin was purified from human plasma as previously reported [31]. The N-terminal fragment of Fn (N29) containing the five N-terminal type I modules, and the gelatin-binding domain (GBD) consisting of four type I modules and two type II modules were isolated following the protocol reported by Zardi *et al* [32]. The recombinant fragment corresponding to residues 607–1265 consisting of the first seven type III modules, the region including the amino acid residues 1266–1908 containing the extra domain B (EDB) plus type III modules 8–13 and the initial region of module 14, and C-terminal fragment 1913–2477 containing one type III module, the IIICS module, three type I modules and the site for interchain disulfide linkage of Fn were purchased from R&D Systems (Minneapolis, MN, USA). Human Fbg and α-elastin, a heterogeneous mixture of soluble peptides obtained from human lung, were obtained from Calbiochem (Merck, Darmstadt, Germany). Fbg was further purified on a gelatin-Sepharose column to remove contaminating Fn. The α-elastin preparation has a molecular weight of 10–60 kDa and runs as a smear on SDS-PAGE.

Bovine, porcine, ovine and canine Fbg as well as bovine serum albumin (BSA) were from Sigma (St Louis, MO, USA). Cultrex® murine sarcoma basement membrane laminin was purchased from Trevigen (Gaithersburg, MD, USA). The synthetic peptide corresponding to the C-terminus of the Fbg γ chain (residues 395–411) and the scrambled peptide were a generous gift by Dr I. Margarit (Novartis Diagnostics & Vaccines, Siena, Italy).

### DNA Manipulation

DNA encoding the SpsD_N1–N3_ subdomains (SpsD_37–519_), the SpsD_N2–N3_ region (SpsD_167–519_) and SpsD_ N2–N3_ lacking C-terminal residues (SpsD_167–498_) were amplified by PCR using chromosomal DNA isolated from *S. pseudintermedius* 326 as template. All oligonucleotides were purchased from Integrated DNA Technologies (Leuven, Belgium). To amplify DNA encoding the N1–N3 domain, primer SpsD N1 forward (5′-GGCGGATCCGCTTCAGAAGT-3′) and SpsD reverse (5′-GAGCTGCAGTTAACTATATCC-3′) were used. To amplify the N2–N3 region, the primers SpsD N2 forward (5′-GATGGATCCGGAACAGATGT-3′) and SpsD reverse were used. To amplify DNA encoding SpsD_167–498_ the primers SpsD N2 forward and SpsD ΔLL reverse (5′-GTAGAAAGCAACACCATTATCCC-3′) were used. Restriction enzyme cleavage sites BamHI and PstI were incorporated at the 5′ ends of the primers to facilitate cloning into the pQE30 expression plasmid (Qiagen, Chatsworth, CA, USA). Restriction enzymes were purchased from New England Biolabs (Hertfordshire, UK). The presence of appropriate DNA sequences was confirmed by sequencing (GATC Biotech, Konstanz, Germany). Amino-acid substitution F288A was introduced by site-directed mutagenesis using overlapping complementary primers (5′-CTTTATCTGGTTTAATAGCTAAGTTCAATGC and 5′ GCATTGAACTTAGCTATTAAACCAGATAAAG-3′. The pQE30 plasmid containing DNA encoding amino acids SpsD_167–519_ was used as template. Products were digested with DpnI to eliminate parental DNA and transformed into *E. coli*. The F228A amino-acid substitution was verified by DNA sequencing (Source Biosciences, Nottingham, UK).

### Expression and Purification of Recombinant Proteins

Recombinant SpsD proteins were expressed from pQE30 plasmid in *E. coli* TOPP3. Overnight starter culture was diluted 1∶50 in LB containing ampicillin (100 µg/ml) and incubated with shaking until the culture reached A_600_ 0.6–0.8. Recombinant protein expression was induced by addition of isopropyl 1-thio-β-D-galactopyranoside (0.1 mM) and continued for 4 h. Bacterial cells were harvested by centrifugation, and frozen at −80°C. Recombinant proteins were purified from cell lysates by Ni^2+^ affinity chromatography on a HiTrap chelating column (GE Healthcare). Protein purity was assessed by SDS-PAGE. Recombinant Bbp_270–599_ [33], ClfA_40–559_ [34], ClfB_201–542_ [26], CNA_30–531_ [35], FnBPB_163–480_ [23] and SasG_50–428_ [36] were each previously expressed with 6 x His N–terminal affinity tags using *E. coli* vectors and purified by Ni^2+^ -chelate chromatography. Mouse cytokeratin 10 (residues 1–570, MK10_1–570_), the N-terminal “head” domain (MK10_1–145_) and the C-terminal “tail” region (MK10_454–570_) proteins were prepared as reported previously [25]. GST fusion proteins containing the human Fbg γ-chain sequence (GST-Fbgγ_395–411_), human Fbg α-chain sequence (GST-Fbgα_316–367_) and the human K10 C-terminal ‘tail’ region (GST-HK10_544–563_) were described previously [26], [37]. GST fusion proteins were purified on a GSTrap FF purification column (GE Healthcare) according to the manufacturer’s instructions.

### SDS-PAGE and Western Blotting

SDS-PAGE was performed with a 10% polyacrylamide gel. The gels were stained with Coomassie Brilliant Blue (BioRad, Hercules, CA, USA). For the Western blot assay, human Fbg and Fn were subjected to SDS-PAGE and then electroblotted onto a nitrocellulose membrane (GE Healthcare). The membrane was treated with a solution containing 5% (w/v) dried milk in PBS, washed, and incubated with SpsD_167–519_ (5 µg/ml) for 1 h at 22°C. Following additional washings with 0.5% (v/v) Tween 20 in PBS (PBST), the membrane was incubated for 1 h with PBST containing an anti-His tag mouse monoclonal antibody (7E8) (2 µg/ml). After several washings in PBST, the membrane was incubated for 45 min with HRP-conjugated rabbit anti-mouse IgG. Finally, the membrane was treated with ECL detection reagents 1 and 2 according to the procedure recommended by the manufacturer (GE Healthcare) and exposed to an X-ray film for 30–60 s.

### Antibodies

Mouse polyclonal antisera against SpsD_37–519_, ClfB_44–542_ and human Fbg were generated by injecting BALB/c mice intraperitoneally five times at 1-week intervals with 50 µg of the purified protein. The antigen was emulsified with an equal volume of complete Freund’s adjuvant for the first immunization, followed by three injections in incomplete adjuvant. The mice were bled, and the sera were tested for reactivity to the purified antigen using ELISA and Western blot. The antibodies were purified by affinity chromatography on Protein A/G-Sepharose columns according to the recommendations of the manufacturer (GE Healthcare). Horseradish peroxidase (HRP)-conjugated rabbit anti-mouse antibody was from Dako (Glostrup, Denmark). 7E8 is an in-house generated monoclonal antibody recognizing recombinant His tagged proteins. This study was carried out in strict accordance with the recommendations in the Guide for the Care and Use of Laboratory Animals of the Italian Ministry of Health according to the European Union guidelines for the handling of laboratory animals. The protocol was approved by the local authorithies (“Comitato Etico per la Sperimentazione Animale” of the University of Pavia, Italy) (Permit Number: 2–2012). The animals were sacrified under lidocaine anesthesia, and all efforts were made to minimize suffering.

### Binding of SpsD Proteins to Extracellular Matrix Proteins by Solid Phase Binding Assay

To screen binding of SpsD to extracellular matrix proteins, 1 µg of human Fbg, human Fn, human lung elastin, murine K10, bovine nasal septum type II collagen, murine sarcoma basement membrane laminin and BSA were coated onto microtiter wells overnight at 4°C in 0.1 M carbonate buffer. The wells were washed, blocked with 2% BSA for 1 h at 22°C and incubated with SpsD_167–519_ or SpsD_37–519_ (17.5 nanomoles/well). Complex formation was detected with mouse anti-His tag antibody 7E8 (0.2 µg/well), followed by addition of peroxidase-conjugated rabbit anti-mouse IgG (1∶1000) (Dako). Binding of SpsD_167–519_ to Fbg from different species in ELISA format was performed by incubating 1 µg of the recombinant bacterial protein with each surface-coated Fbg type (1 µg/well) and detecting the bound protein as reported above.

### Surface Plasmon Resonance (SPR)

All experiments were performed at 25°C on a BIAcore X instrument (Pharmacia Biosensor AB, Uppsala, Sweden). Purified human and canine Fbg, α-elastin or human Fn were covalently immobilized on the dextran matrix sensor chip surface (CM5 chip) by using a protein solution (30 µg/ml in 50 mM sodium acetate buffer, pH 5.5 for human and canine Fbg or 50 mM sodium acetate buffer, pH 4.0 for human Fn) in a 1∶1 dilution with N-hydroxy-succinimide and N-ethyl-N’-(3-dimethylaminopropyl)-carbodiimide hydrochloride. The excess active groups on the dextran matrix were blocked with ethanolamine (1 M), pH 8.5 [38]. On another flow cell, the dextran matrix was treated as described above but without any ligand to provide an uncoated reference flow cell. The running buffer used was PBS containing 0.005% (v/v) Tween 20. Increasing concentrations of SpsD proteins were passed in succession over the ligand and reference surfaces without regeneration at the flow rate of 5 µl/min in running buffer.

To perform SPR experiments with GST-HK10_544–563_, goat anti-GST antibody IgG (30 µg/ml) (GE Healthcare) was diluted in 10 mM sodium acetate buffer, pH 5.0, and immobilized on carboxy-derivatized sensor chip. GST-HK10_544–563_ (500 nM) was passed over a flow cell, while GST alone was passed over the other flow cell to provide a reference surface. Increasing concentrations of SpsD proteins were flowed over the surface of both flow cells without regeneration at a rate of 5 µl/min.

Sensorgrams from three sets of data for each of the protein concentrations were collected and averaged for each ligand. All of the sensorgram data presented were subtracted from the corresponding data from the reference flow cell. The data were analyzed using the BIAevaluation software version 3.0. A plot of the level of binding (response units) at equilibrium against concentration of ligand was used to determine the *K_D_*.

### Inhibition of the SpsD-Fbg Interaction

A solution of Fbg (10 µg/ml) was used to coat microtiter wells overnight at 4°C. The wells were washed three times with PBST and blocked with 2% (w/v) BSA in PBS for 1 h at 22°C. The wells were washed and 100 µl SpsD proteins (10 µg/ml) added after preincubation for 1 h at 22°C with a range of concentrations of a synthetic peptide comprising the 17 C-terminal residues of the γ chain of Fbg or a scrambled peptide. The plates were incubated for 1 h at 22°C and bound protein detected by incubation with anti-His tag mouse 7E8 monoclonal antibody (0.2 µg/well) for 1 h at 22°C, followed by horseradish peroxidase-conjugated rabbit anti-mouse IgG serum. The percentage inhibition was calculated relative to the level of Fbg-bound protein detected in the absence of the inhibitor peptide.

### Inhibition of Fibrin Clot Formation

Thrombin-catalyzed Fbg clotting was evaluated by incubating 150 µl of 3 µM Fbg in 20 mM Tris/HCl, pH 7.4 containing 200 mM NaCl and 5 mM CaCl_2_ with the indicated concentrations of SpsD_167–519_, Bbp_270–569_ or BSA and 50 µl of thrombin (1.0 NIH unit/ml). Clot formation was measured as a change in turbidity with time related to the amount of the scattered light at 350 nm in a Jasco, V-630 spectrophotometer (Jasco, Tokyo, Japan).

### Inhibition of Platelet Aggregation

300 µl (9×10^7^) washed platelets were preincubated in a Born aggregometer (Chrono-Log Havertown, PA, USA) with either buffer or the indicated concentrations of SpsD_167–519_ or region A of ClfA or CNA in the presence of 200 µg/ml Fbg and 1 mM CaCl_2_ at 37°C for 5 min. Then 10 µM ADP was added to trigger platelet activation and aggregation. The rate and extent of platelet aggregation was detected as the percentage of light transmission and presented as aggregation tracings.

### SpsD Binding to Immobilized Fn Fragments by ELISA and Dot Blot

Fn fragments were used to identify binding sites of SpsD. Fn (1 µg/well) and Fn fragments (250 ng/well) were immobilized onto microtiter wells and then incubated with indicated concentrations of SpsD_167–519_. Bound protein was detected using an anti-SpsD_37–519_ mouse antibody followed by HRP-conjugated rabbit anti-mouse IgG. For dot blot experiments, different amounts of Fn and Fn fragments (1000- 62.5 ng) were dotted onto a nitrocellulose membrane, probed with SpsD_167–519_ (1 µg/ml) and complexes detected as described above.

## Results

### Region A of SpsD

The sequence of the entire *spsD* gene of *S. pseudintermedius* ED99 is known. Here we describe the A domain of SpsD from strain 326 which was obtained following PCR amplification of genomic DNA. The A domains of SpsD from ED99 and 326 have the same number of residues and are 80% identical. They are most closely related to the A domain of FnBPB (40% and 42% identity to isoform I, respectively) [39] and they have 23% and 27% identity respectively to the A domain of ClfA from *S. aureus* Newman [40]. A 3D molecular model based on the crystal structure of ClfA allowed us to predict the coordinates of subdomains N1, N2 and N3 (Figure 1).

The SpsD A domain (N1N2N3 region) comprising residues 37–519 (SpsD_37–519_) and the putative minimum ligand binding truncate comprising residues 167–519 (N2N3, SpsD_167–519_) were cloned and expressed with 6 x His affinity tags.

### Binding of Recombinant SpsD to Extracellular Matrix Proteins

**Figure 2:**
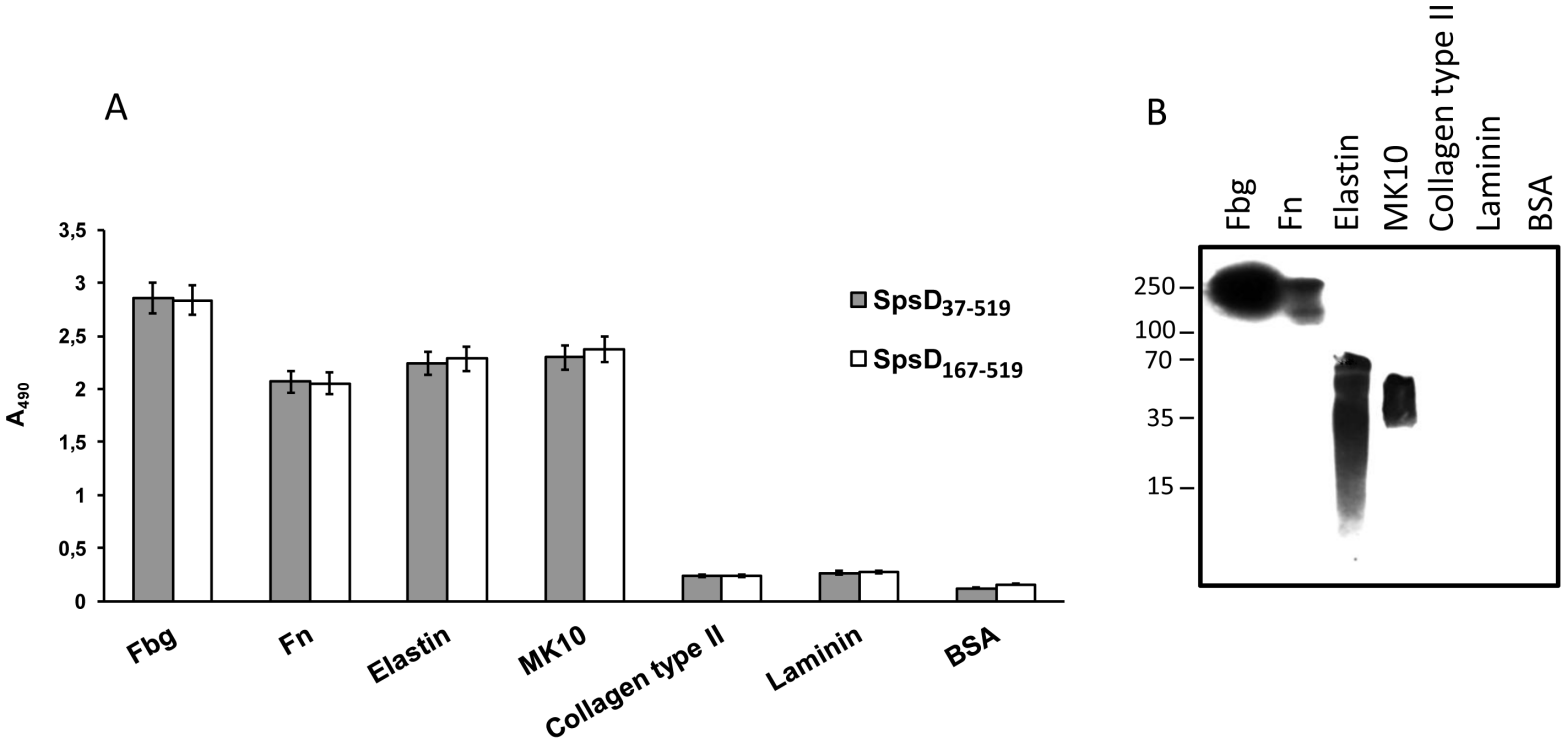
Binding of SpsD proteins to extracellular matrix or plasma proteins. (A), ligands were coated onto the surface of microtiter plates (1 µg/well) and incubated with 17.5 nanomoles of SpsD_37–519_ or SpsD_167–519_, followed by mouse anti-His IgG and HRP-conjugated rabbit anti-mouse IgG. *A*, absorbance. (B), purified, unreduced human Fbg, Fn, α-elastin, mouse K10, collagen type II, laminin and BSA were subjected to SDS-PAGE, transferred to nitrocellulose filter and then incubated with SpsD_167–519_, followed by incubation with mouse anti-SpsD_37–519_ and HRP-conjugated rabbit anti-mouse antibodies. Molecular mass markers are indicated (kilodaltons).

Previous work has demonstrated that SpsD can promote bacterial adhesion to Fbg, Fn and K10 [11]. Here we sought to determine if the ligand binding ability resides in the N-terminal domain of the protein. An ELISA type ligand-binding assay was used to study the interaction of the recombinant SpsD proteins with surface-coated extracellular matrix proteins. SpsD_37–519_ and SpsD_167–519_ bound to immobilized Fbg, Fn and K10, as well as to α-elastin, while no interaction was observed with collagen, laminin or BSA (Figure 2A). The interaction of Fbg, Fn, K10 and α-elastin with the recombinant SpsD_167–519_ protein was confirmed by Western ligand blotting where host proteins were electroblotted onto a nitrocellulose filter, probed with SpsD_167–519_ and complex formation detected with a monoclonal antibody targeting the His-tag. Conversely, no interaction was observed when SpsD_167–519_ was incubated with membrane-bound collagen type II, laminin or BSA (Figure 2B). Identical results were obtained with SpsD_37–519_ (data not shown). Together these results suggest that the minimal region of SpsD required for binding to Fbg, Fn, K10 and elastin is the N2N3 subdomain. The absence of signal when Fbg, Fn, α-elastin and K10 were tested for binding to His-tagged *S. aureus* MSCRAMM SasG_50–428_ either in ELISA or Western blotting, further demonstrates the specificity of SpsD_167–519_ interaction with host ligands (data not shown).

**Figure 3:**
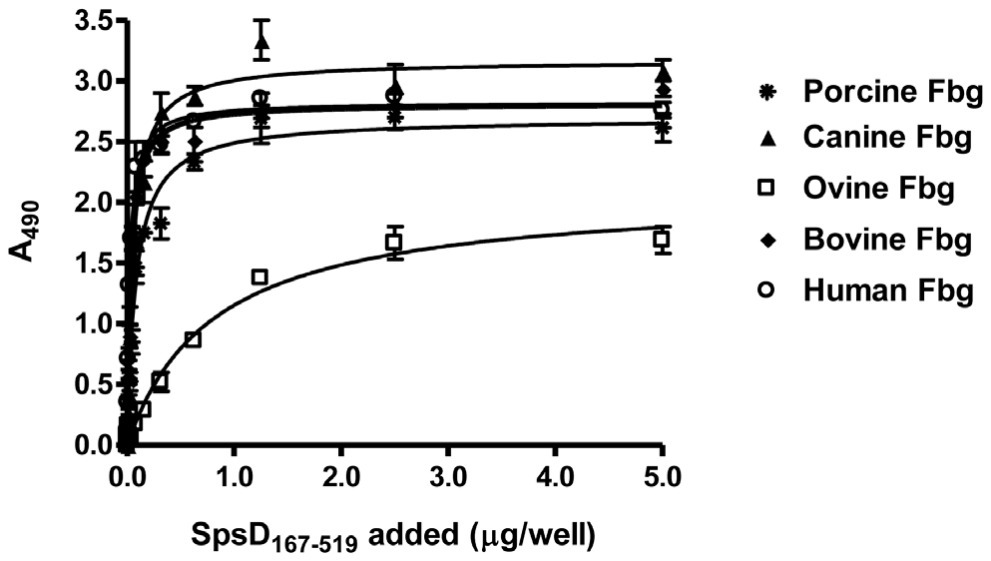
Dose-dependent binding of SpsD_167–519_ to Fbg from different species. Microtiter wells were coated with human, canine, ovine, bovine and porcine Fbg overnight. The wells were probed with SpsD_167–519_ followed by incubation with mouse anti-His-IgG and HRP-conjugated rabbit anti-mouse IgG. Values represent the means of triplicate samples ± S.E. This experiment was performed three times with similar results.

Next we determined the ability of SpsD_167–519_ protein to recognize Fbg molecules obtained from different animals. Increasing amounts of SpsD_167–519_ were added to surface-coated human, porcine, canine, bovine and ovine Fbg and the binding was measured. SpsD_167–519_ bound to each of the different types of Fbg with similar affinities with exception of the ovine protein (Figure 3).

### SpsD Binds the C-terminus of the γ-chain of Fbg

**Figure 4:**
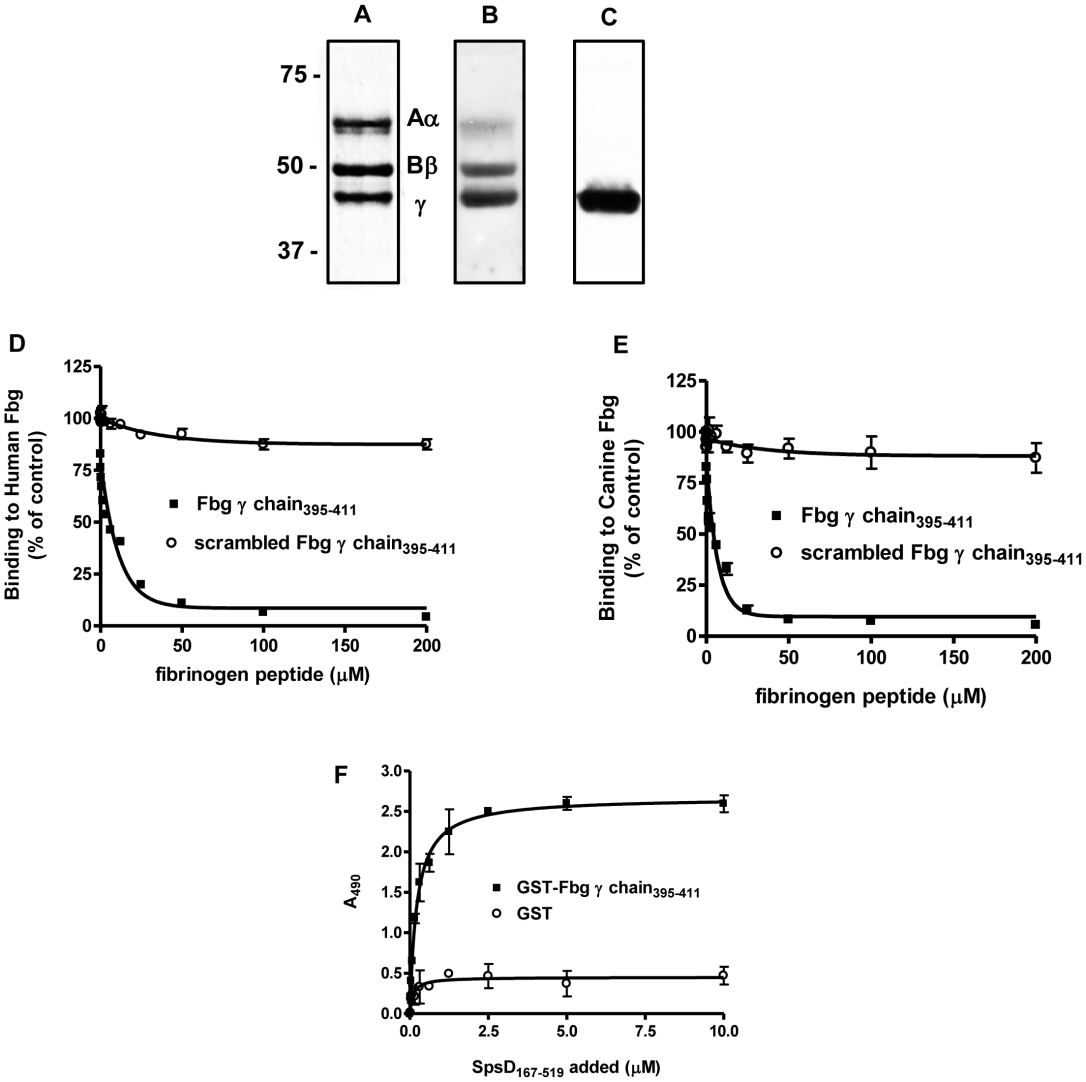
SpsD recognizes Fbg γ chain. (A), human Fbg was subjected to SDS-PAGE in reducing conditions and then stained with Coomassie Blue. (B), Fbg was subjected to SDS-PAGE in reducing conditions, transferred to a nitrocellulose membrane and Fbg chains detected by incubation of the membrane with anti-mouse Fbg IgG and then HRP-conjugated rabbit anti-mouse IgG. (C), Fbg was subjected to SDS-PAGE under reducing conditions, electroblotted onto nitrocellulose filter and probed with SpsD_167–519_ followed by incubation with mouse anti-SpsD IgG and HRP-conjugated rabbit anti-mouse IgG. (D) and (E), effect of the Fbg C-terminal γ chain peptide on the binding of SpsD_167–519_ protein to immobilized Fbg. Surface-coated human (D) or canine (E) Fbg was incubated with SpsD_167–519_ in the presence of increasing concentrations of the 17- mer synthetic peptide of the γ chain. The effect of a scrambled peptide on Fbg-SpsD_167–519_ interaction is also reported as control; the assay was performed as in (C). (F), dose-dependent binding of SpsD_167–519_ protein to immobilized GST fused to the Fbg C-terminus of γ chain. Microtiter wells were coated with GST-Fbg γ chain and probed with increasing concentrations of SpsD_167–519_. Complex formation was detected by addition of mouse anti- 6-His IgG followed by HRP-conjugated rabbit anti-mouse IgG. Values are the means ± S.E of triplicate wells and are representative of one experiment. This experiment was performed three times with similar results.

Western ligand blotting was employed to determine if SpsD bound to the α-, β- or γ- chain of Fbg. Human Fbg was reduced with dithiothreitol and the three chains separated by SDS-PAGE (Figure 4A). Efficient transfer to a control nitrocellulose membrane was shown by probing with anti-Fbg IgG (Figure 4B). Another membrane was incubated with recombinant His-tagged SpsD_167–519_ and when probed with a monoclonal antibody recognizing the His-tag only the γ-chain reacted (Figure 4C).

ClfA, FnBPA and FnBPB are known to bind to a short sequence at the extreme C-terminus of the γ-chain of Fbg while ClfB binds to repeat number 5 of the α chain. To determine if SpsD bound the same γ chain residues as ClfA, the ability of a 17 residue synthetic peptide comprising the C-terminal residues of the Fbg γ-chain to inhibit binding of SpsD_167–519_ to immobilized Fbg was tested. The peptide strongly inhibited the binding of SpsD to human and canine Fbg dose-dependently while a scrambled peptide had no effect (Figure 4D and 4E).

Finally, direct binding of SpsD to the Fbg γ-chain peptide coupled to GST that had been immobilized on the surface of an ELISA plate was tested. As shown in Figure 4F, SpsD bound dose-dependently and saturably to GST-Fbgγ_395–411_ with a half maximum binding concentration of 0.32±0.02 µM but not at all to the GST control. Thus, SpsD binds to the same C-terminal Fbg γ-chain residues as ClfA and the FnBPs.

### Region A of SpsD Interferes with Fibrin Clot Formation and Platelet Aggregation

**Figure 5:**
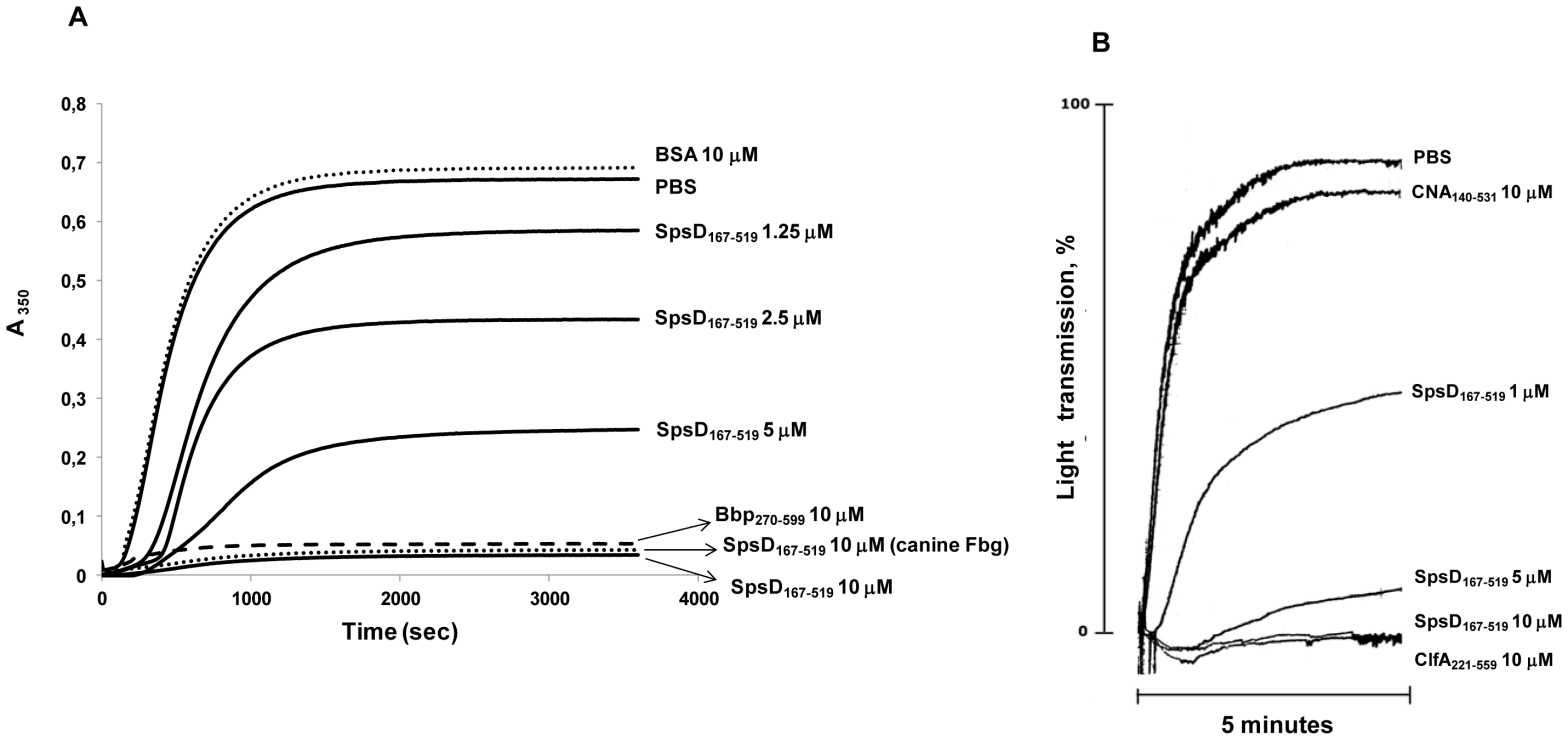
SpsD interferes with Fbg biological activities. (A), SpsD protein inhibits fibrin clot formation. Increasing amounts (1.25–10 µM) of SpsD_167–519_ were preincubated with human Fbg prior to the addition of α-thrombin. Polymer formation was measured as a change in turbidity at 350 nm with time. The action of 10 µM Bbp_270–599_ and BSA are shown as a positive and negative controls, respectively. The effect of 10 µM SpsD_167–519_ on canine fibrin clot formation is also reported. (B), SpsD protein inhibits ADP-induced platelet aggregation. Washed human platelets (9×10^7^) were preincubated in an aggregometer with the indicated concentrations of SpsD_167–519_, ClfA_40–560_ and CNA_30–531_ in the presence of 200 µg/ml Fbg and 1 mM CaCl_2_ at 37°C for 5 min and then added with 10 µM ADP. Platelet aggregation is expressed as the percentage of light transmission. The experiments have been repeated at least three times with similar results.

Several adhesins not only interact with the target molecule in the host but also neutralize and even disrupt the biological function of the target. For example, ClfA, although it does not interfere with catalytic activity of thrombin, by binding to the C-terminus of γ chain it affects fibrin clot formation by abrogating fibrin monomer polymerization and cross-linking of Fbg γ chains [41]. Therefore, we examined the effect of SpsD on fibrin clot formation. Human Fbg was preincubated with different concentrations (1.25–10 µM) of SpsD_167–519_ prior to the addition of thrombin. In these conditions SpsD inhibited coagulation as effectively as Bbp_270–599_, a recently identified Fbg-binding protein from *S. aureus* [33] (Figure 5A). Conversely, no effect was observed when Fbg was preincubated with 10 µM BSA. Consistent with the indication that SpsD recognizes the C-terminus of the γ chain of canine Fbg, we also observed that preincubation of canine Fbg with 10 µM SpsD resulted in complete inhibition of clot formation.

It is well known that the C-terminus of Fbg γ chain contains the platelet integrin GPIIb/IIIa recognition sequence (QAGDV^411^) [42] and that a monoclonal antibody that recognizes the γ chain C-terminus of Fbg exerts inhibitory effects on platelet aggregation [43]. Thus, SpsD was tested for its impact on ADP-induced platelet aggregation. As depicted in Figure 5B, ADP-stimulated platelet aggregation was inhibited in a dose-dependent manner by SpsD. In contrast, the collagen-binding domain of the *S. aureus* adhesin CNA (CNA_30–531_), did not affect the process. Together these data demonstrate that SpsD could act as a modulator of thrombus formation.

### Binding Sites in Fibronectin for SpsD

**Figure 6:**
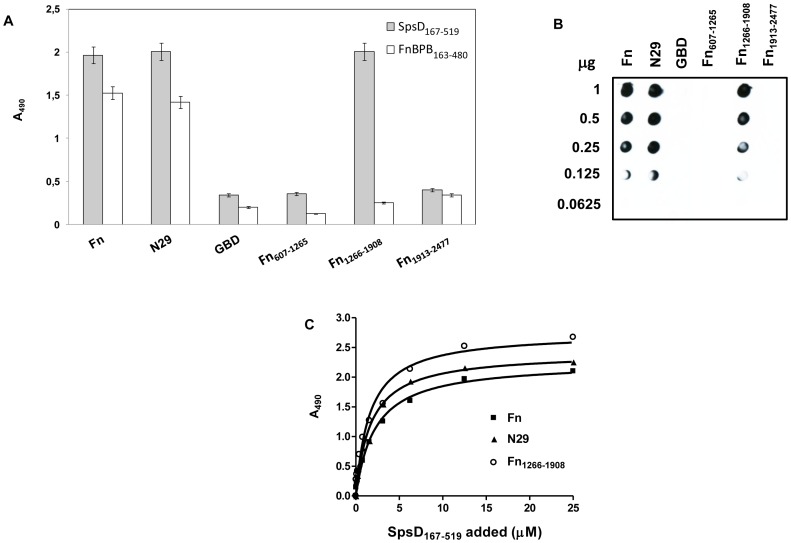
Localization of SpsD binding sites in Fn. (A), native Fn, the N29, the GBD, the recombinant fragments Fn_607–1263_, Fn_1266–1908_ and the C-terminal region Fn_1913–2477_ of Fn were immobilized onto a microtiter plate and then incubated with of SpsD_167–519_. Bound protein was detected by addition to the wells of anti-SpsD_37–519_ mouse antibody, followed by HRP-conjugated rabbit anti-mouse antibody. Binding of FnBPB_163–480_ to Fn and its fragments reported as control was detected by addition to the wells of anti-FnBPB_163–480_ rabbit IgG, followed by HRP-conjugated goat anti-rabbit antibody. (B), different amounts of Fn and Fn fragments (1000-62.5 ng) were dotted onto a nitrocellulose membrane, probed with SpsD_167–519_ (1 µg/ml) and complexes detected by addition of mouse anti-SpsD_37–519_ IgG, followed by HRP-conjugated rabbit anti-mouse IgG. (C), surface-coated Fn, the N29 fragment and the region including the aa residues 1266–1908 were incubated with increasing concentrations of SpsD_167–519_ and complex formation detected as reported in (A). Results shown in panels A and C are the mean values of triplicate samples ± S.E. The experiments were repeated three times with similar results.

To locate the binding site(s) in Fn for the A domain of SpsD, the binding of SpsD_167–519_ to the Fn N29 domain, the gelatin binding domain and the segments spanning residues 607–1265, 1266–1908 and 1913–2477 was tested. Native Fn and the purified fragments were immobilized onto microtiter wells, probed with SpsD_167–519_ and complex formation detected with anti-SpsD IgG. A strong signal was observed when SpsD_167–519_ was incubated with full length Fn, the N29 fragment and the segment spanning the residues 1266–1908 (Fn_1266–1908_), while the other fragments failed to bind (Figure 6A). A similar assay was performed with FnBPB_163–480_ and confirmed its ability to bind to full length Fn and the N29 fragment and not to the C-terminal Fn_1266–1908_ fragment [23]. The binding of SpsD to the two different regions of Fn was confirmed by incubating the Fn fragments with SpsD_167–519_ in a dot blot assay (Figure 6B). Finally, as shown in Figure 6C, binding of SpsD_167–519_ to both the N29 and the Fn_1266–1908_ fragment was concentration-dependent and saturable. The affinity for each domain appeared to be similar with half maximum binding concentrations of 2.21±0.30 µM for N-terminal domain and 1.97±0.3 µM for Fn_1266–1908_.

### SpsD Binds to the K10 Tail Region

**Figure 7:**
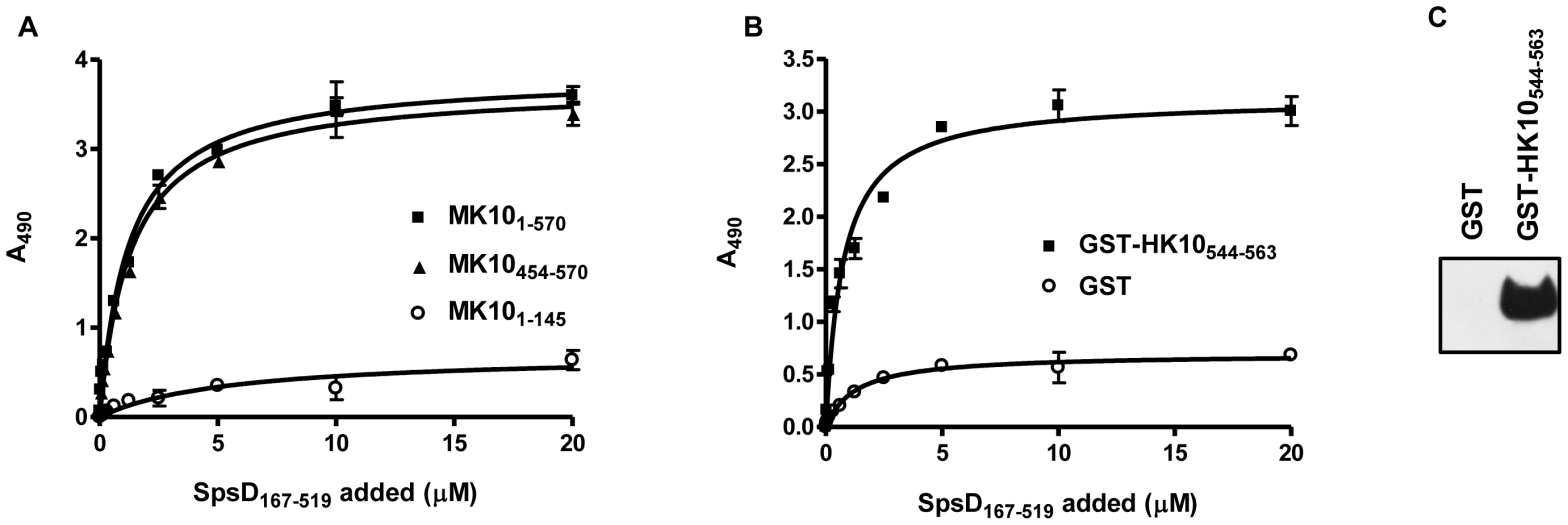
Binding of SpsD_167–519_ to immobilized cytokeratin 10 proteins. (A), SpsD_167–519_ was incubated in wells coated with recombinant mouse K10_1–570_, K10_1–145_ and K10_454–570_. (B), SpsD_167–519_ was added to ELISA wells coated with GST-human K10_544–563_, (HK10_544–563_); or GST alone. Bound SpsD_167–519_ was detected by addition of mouse anti-SpsD_37–519_ IgG, followed by HRP-conjugated rabbit anti-mouse IgG. (C), GST-HK10_544–563_ or GST alone were subjected to SDS-PAGE, electroblotted onto nitrocellulose filter and probed with SpsD_167–519_ followed by incubation with mouse anti-SpsD IgG and HRP-conjugated rabbit anti-mouse IgG.

ClfB binds to the C-terminal tail region of K10 [25]. To determine if SpsD binds to the same domain, recombinant proteins comprising full length murine K10 along with the N-terminal head and C-terminal tail regions, were immobilized into microtitre wells and tested for their ability to support SpsD binding. SpsD_167–519_ bound to full length K10 and the tail region dose-dependently and saturably and with half maximal binding concentrations of 1.02±0.25 and 1.2±0.1 µM, respectively (Figure 7A).

ClfB is known to bind to glycine (G)-serine (S)-rich domains within the omega loop regions in the C-terminal tail of K10, in loricrin and in the α-chain of Fbg [26]. To determine if SpsD could bind to the same sequences, recombinant SpsD_167–519_ was tested in solid phase binding assays with GST-HK10_544–563_. The SpsD protein bound dose-dependently and saturably but failed to bind to the GST (Figure 7B). This interaction was confirmed by Western blot (Figure 7C). However, in contrast to ClfB, SpsD did not interact with Fbg α chain (Figure S1). Consistent with this, GST-Fbgα_316–367_ inhibited binding of ClfB, but not that of SpsD_167–519_, while GST-Fbgγ_395–411_ blocked the interaction of SpsD_167–519_ but not that of ClfB with Fbg (Figure S2). Thus SpsD bound the same GS-rich omega loop in K10 as ClfB but surprisingly failed to recognize the Fbg α chain.

### Measurements of Dissociation Constants of SpsD for Host Ligands by Surface Plasmon Resonance

**Figure 8:**
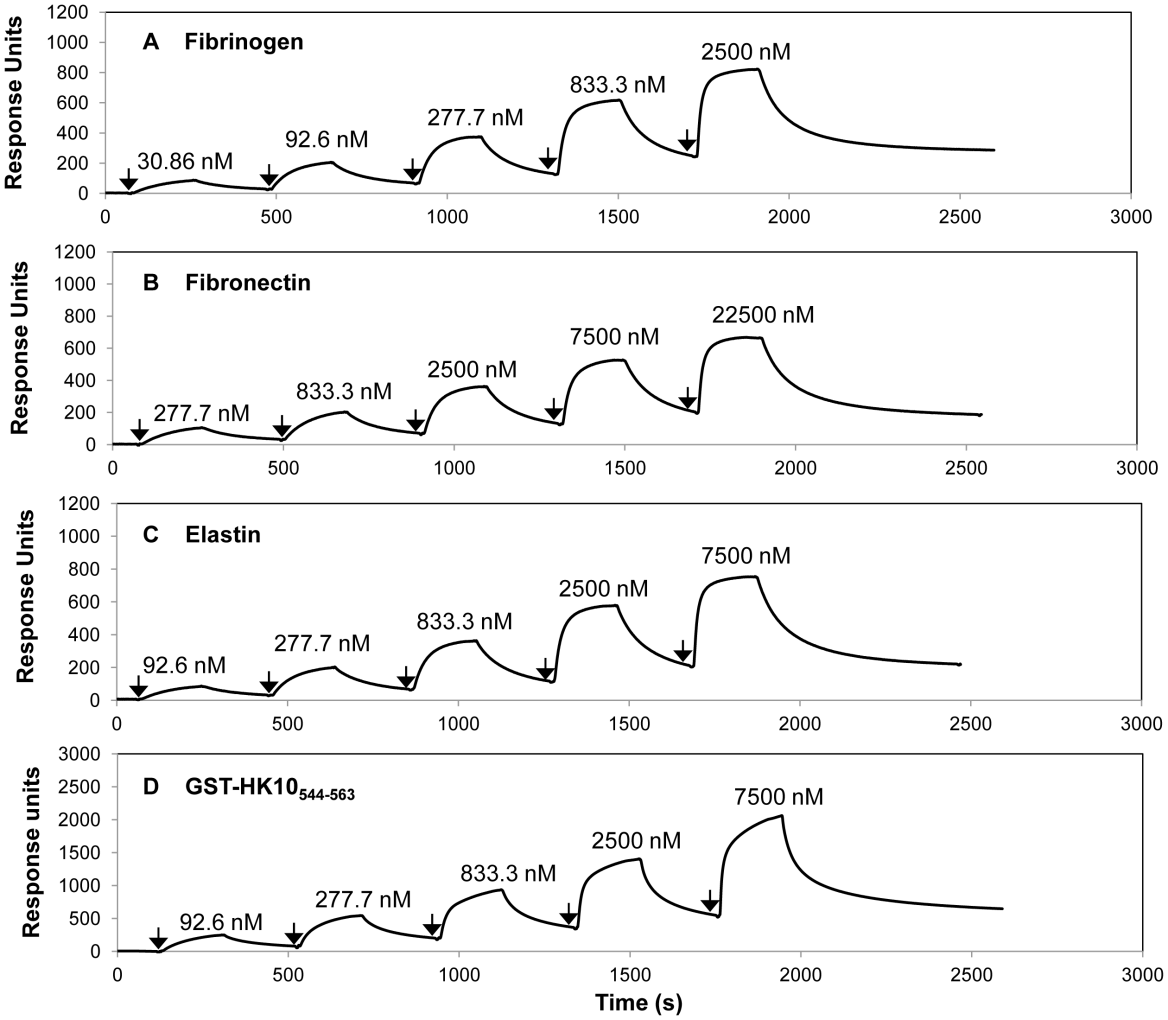
Surface Plasmon Resonance analysis of the interaction of SpsD_167–519_ with Fbg, Fn, α-elastin and cytokeratin 10. Human Fbg (A), Fn (B) and α-elastin (C) were immobilized onto the surface of dextran chips. GST-human K10_544–563_, (D) was captured onto a sensor chip coated with goat anti-GST IgG. Representative sensorgrams display binding of SpsD_167–519_ to and dissocation from Fbg, Fn, α-elastin and GST-human K10_544–563_, in a single cycle kinetics assay. Binding was measured as response units (RU) against time. The affinities were calculated from curve fitting to a plot of the RU values against concentrations of SpsD. Arrows indicate the time at which SpsD_167–519_ was injected. The data shown is representative of 3 individual experiments.

Binding of SpsD_167–519_ to human Fbg, Fn, α-elastin or GST-HK10_544–563_ immobilized on a sensor chip was analyzed by SPR using BIAcore X. Increasing concentrations of SpsD_167–519_ were passed over the surface of each ligand-coated chip: in these conditions SpsD_167–519_ bound to the ligand in a dose dependent-manner. From analysis of the equilibrium binding data, the dissociation constant (*K_D_*) for the interaction between SpsD_167–519_ and Fbg was determined to be 0.360±0.032 µM (Figure 8A and Table 1). SpsD_167–519_ showed a dissociation constant value for canine Fbg comparable to that reported for human Fbg (Table 1).

**Table 1:** Summary of *K_D_* ± SE values of SpsD proteins for extracellular matrix components.

	*K_D_* (µM) ± SE	
	SpsD_37–519_	SpsD_167–519_
Human Fbg	0.380±0.035	0.360±0.032
Canine Fbg	0.367±0.055	0.341±0.056
Fn	2.350±0.710	2.157±0.702
α-Elastin	1.200±0.220	1.020±0.150
GST-HK10_544–563_	0.906±0.060	0.840±0.032

SpsD_167–519_ bound Fn with a *K_D_* of 2.16±0.70 µM, indicating that the affinity of the recombinant SpsD protein for Fn was lower than that exhibited for Fbg (Figure 8B and Table 1). The *K_D_* values of SpsD_167–519_ for α-elastin and GST-HK10_544–563_ were 1.02±0.15 and 0.84±0.032 µM, respectively (Figure 8, panels C and D and Table1). Very similar *K_D_* values were obtained when SpsD_37–519_ was passed over sensor chips coated with these proteins (Table 1).

### Ligand Binding by SpsD_167–519_ Mutants

**Figure 9:**
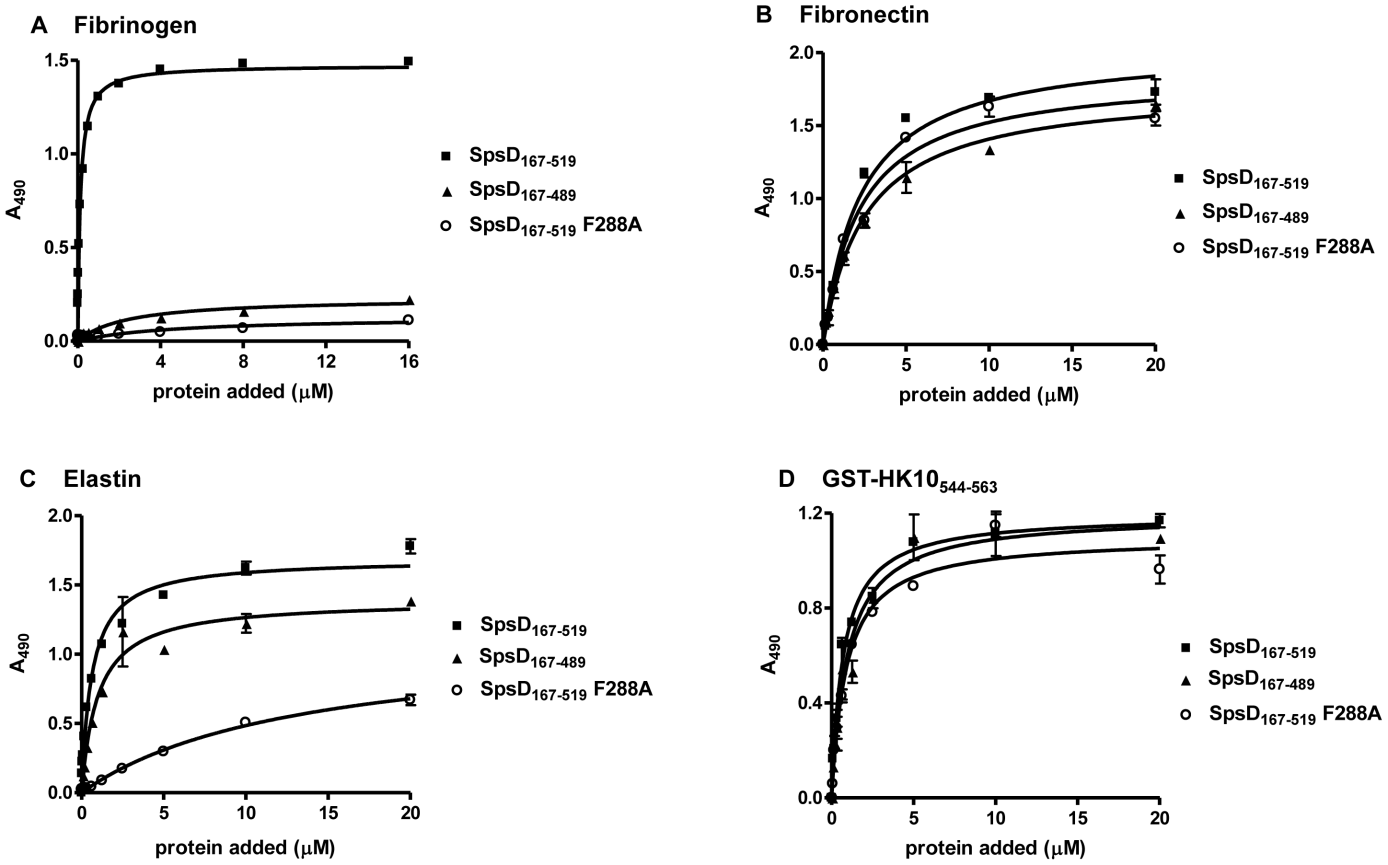
Binding of SpsD_167__–519_, SpsD_167__–519_ F288A and SpsD_167__–519_ latch mutant to immobilized Fbg, Fn, α-elastin and K10. Microtiter wells were coated with human Fbg (A), Fn (B), α-elastin (C) and K10 (D) in a bicarbonate buffer overnight. The wells were probed with increasing concentrations of SpsD proteins followed by incubation with mouse anti SpsD_37–519_ IgG and HRP-conjugated rabbit anti-mouse IgG. Values are the means ± S.E of triplicate wells and are representative of one experiment. This experiment was performed three times with similar results.

One goal of this work was to determine whether the N2N3 subdomains of SpsD bind Fbg by the same mechanism as the A domains of ClfA and FnBPs. Our previous work demonstrated that the C-terminal residues of the N3 subdomain of FnBPB were crucial for its interaction with Fbg [23]. As SpsD interacts with the Fbg γ-chain with a high affinity, it was proposed that the C-terminal 20 residues of the N3 subdomain of SpsD form the locking and latching segment and play a similar role in the interaction of SpsD with Fbg. To test this, a recombinant truncate of the SpsD_167–519_ protein, which lacked the predicted locking and latching segment (SpsD_167–498_: ΔLL, Figure 1), was expressed and its ability to bind to immobilized Fbg was analysed by ELISA. No detectable interaction was observed when increasing concentrations of SpsD_167–499_ were incubated with Fbg-coated microtiter wells (Figure 9A), while a small but remarkable reduction in elastin binding was noticed (Figure 9C). In contrast, no reduction in binding was observed when increasing amounts of SpsD_167–499_ were incubated with surface-coated Fn or K10 (Figure, 9 panels B and D). To investigate further if SpsD binds to the ligands at same or different sites, an amino acid substitution was introduced in SpsD_167–519_ at residue F288. F288 is predicted to be located within the ligand binding trench and is in an equivalent position to the crucial residues F314 of FnBPB and Y338 of ClfA. SpsD F288A binding to Fbg and elastin was greatly reduced showing that F288 is indeed important for ligand binding and supports the notion that both Fbg and elastin bind to the same or overlapping regions of SpsD. Conversely, a small reduction was observed when SpsD F288A was tested for binding to Fn or K10.

## Discussion

The ability of *S. aureus* cells to adhere to components of the extracellular matrix is promoted by several distinct surface proteins called MSCRAMMs. The mechanistic basis of many of the interactions has been very well characterized by structural analysis and by probing function with appropriate variant mutant proteins. By molecular modelling and mutagenesis it is possible to infer/predict that proteins with similar structural organization bind their ligands by the same mechanism as those that have been investigated in detail.

The A domain of ClfA is the archetype of a family of proteins that fold into three subdomains called N1, N2 and N3, the last two of which are the absolute minimum required for ligand binding by the “dock, latch and lock (DLL)” mechanism [18]. The A domain of ClfA binds to the γ-chain of Fbg by DLL and it does not interact with Fn, elastin or K10 [44]–[46]. In contrast, the A domain of ClfB binds by DLL to the α-chain of Fbg and also to K10. In both cases the DLL mechanism was shown by X-ray crystal structure and supported by the loss of binding by mutants that had substitutions in the ligand binding trench (trench mutants) or which lack the C-terminal lock-latch domain (LL mutants) [24].

The Fn binding proteins FnBPA and FnBPB are composed of two distinct ligand binding domains. Their A domains are most closely related to ClfA and bind to the Fbg γ-chain [16], [23]. In contrast to ClfA, both FnBPA and FnBPB also bind to elastin [21] and in the case of FnBPB, Fn [23]. Binding to Fbg and elastin most likely occurs by DLL as indicated by studies with mutant proteins [17]. Ten or 11 tandemly repeated domains at the C-termini of FnBPA and FnBPB bind to Fn with high affinity by the tandem β zipper mechanism [29].

The SpsD protein of *S. pseudintermedius* was previously shown to promote bacterial adhesion to Fbg, Fn and K10 [11], [15]. In this study we have dissected the SpsD protein and have investigated how a single protein can bind to such a disparate array of ligands. Bioinformatic analysis predicted that the N-terminal region folded into a typical A domain composed of subdomains N1 N2 and N3 and was most closely related in sequence identity to FnBPB [11]. Although there is a repeat domain at the C-terminus of SpsD, the individual repeats are shorter than, and bear no similarity to, the Fn-binding repeats of FnBPs [11].

The recombinant A domain of SpsD was shown here to bind to the γ-chain of Fbg (and not to the α-chain), to K10, to Fn and also to elastin. There are intriguing similarities to and differences from the MSCRAMM A domains of *S. aureus*.

To investigate if the DLL mechanism is involved in ligand binding, ΔLL and trench mutants of the N2N3 construct were expressed and purified. Binding to Fbg γ-chain was completely abrogated in the SpsD ΔLL and trench mutants indicating that binding to this ligand occurred by DLL. However SpsD also bound to K10 which is a defining property of ClfB, while ClfB binds to the α-chain and not to the γ-chain of Fbg [27]. The ligand peptide sequences that bind to the trench are completely dissimilar. Indeed structural biology showed that the Fbg α-chain peptide and the K10 omega loop peptide bound to the same part of the ligand binding trench of ClfB and a consensus motif was identified [24]. Therefore it was intriguing to discover that the ΔLL and trench mutants of SpsD bound to K10 with the same affinity as the wild-type while similar mutants of ClfB were previously shown to be defective in binding to both ligands [24]. We can only conclude that SpsD binding to K10 does not occur by DLL and propose a distinct K10 binding site in N2N3.

Like its closest relative FnBPB, SpsD also binds to elastin. This most likely occurs by DLL because the trench and the ΔLL mutants were both defective in binding to this ligand. Although a similar investigation was not carried out with FnBPB, detailed studies with the A domain of FnBPA supported the DLL mechanism for elastin binding [17]. This confirms the close relationship of the A domain of SpsD to those of the FnBPs.

It was not clear if the binding of FnBPB A domain to Fn occured by DLL because the ΔLL truncate bound Fn with the same affinity as the wild-type while a trench mutant was defective in both Fbg and Fn binding. It seemed that the Fn binds to FnBPB in the trench region but that lock latch aspect of DLL is not involved [23]. The SpsD A domain shares with FnBPB the ability to bind Fn but with the difference that it bound to two distinct domains within the host protein while FnBPB only recognized the N29 fragment [23]. This might explain why both the SpsD ΔLL and trench mutants bound Fn while the ΔLL mutant of FnBPB was defective.

A domains can be stitched onto distinct “stalks” (B repeats, Sdr repeats, Fn-binding repeats, or to a complete different sequence in SpsD). The DLL binding mechanism in N2N3 regions has evolved to binds to different ligands. Indeed even the orientation of a peptide ligand can vary [24]. Many have acquired the capacity to bind ligands by mechanisms other than DLL although the binding of a large folded ligand might occlude the trench region and block DLL mechanism of binding to other ligands.

In conclusion, SpsD appears to be the most promiscuous staphylococcal adhesin so far identified and possibly is a factor involved in *S. pseudintermedius* colonization of the host. In future studies the three-dimensional structure of the apo-protein and ligand bound SpsD may provide molecular details on these multiple interactions and give additional insights to better understand the role played by this microganism in human and animal pathogenesis.

## Supporting Information

Figure S1 **Binding of SpsD_167–519_ or ClfB_201–542_ to Fbg and Fbg α chain.** Increasing concentrations of SpsD_167–519_ (A) or ClfB_201–542_ (B) were incubated in wells coated with 500 ng/well human Fbg, GST in fusion with the α chain peptide (aa 316–367) or GST alone. Bound ligand was detected by addition of mouse anti-SpsD_37–519_ IgG or anti-ClfB_45–542_ IgG followed by HRP-conjugated rabbit anti-mouse IgG. Results shown in the panels are the mean values of triplicate samples ± S.E. The experiments were repeated three times with similar results.
(TIF)

Figure S2 **Effect of Fbg recombinant chains on the binding of SpsD_167–519_ or ClfB_201–542_ to surface-coated Fbg.** 500 ng SpsD_167–519_ (A) or ClfB_201–542_ (B) mixed with increasing amounts of GST-tagged α chain, GST in fusion with the C-terminus of the γ chain, or GST alone were added to and incubated with microtiter wells coated with Fbg (500 ng/well). Bound ligand was detected by addition of mouse anti-SpsD_37–519_ IgG or anti-ClfB_45–542_ IgG followed by HRP-conjugated rabbit anti-mouse IgG. Results shown in the panels are the mean values of triplicate samples. Error bars show the standard deviation. The experiments were repeated three times with similar results.
(TIF)
