# Supplementary figures and images for: Molecular Characterization of the Multiple Interactions of SpsD, a Surface Protein from Staphylococcus pseudintermedius, with Host Extracellular Matrix Proteins
Source: PLoS One. 2013 Jun 21;8(6):e66901. doi: 10.1371/journal.pone.0066901 (PMC3689669; doi:10.1371/journal.pone.0066901)

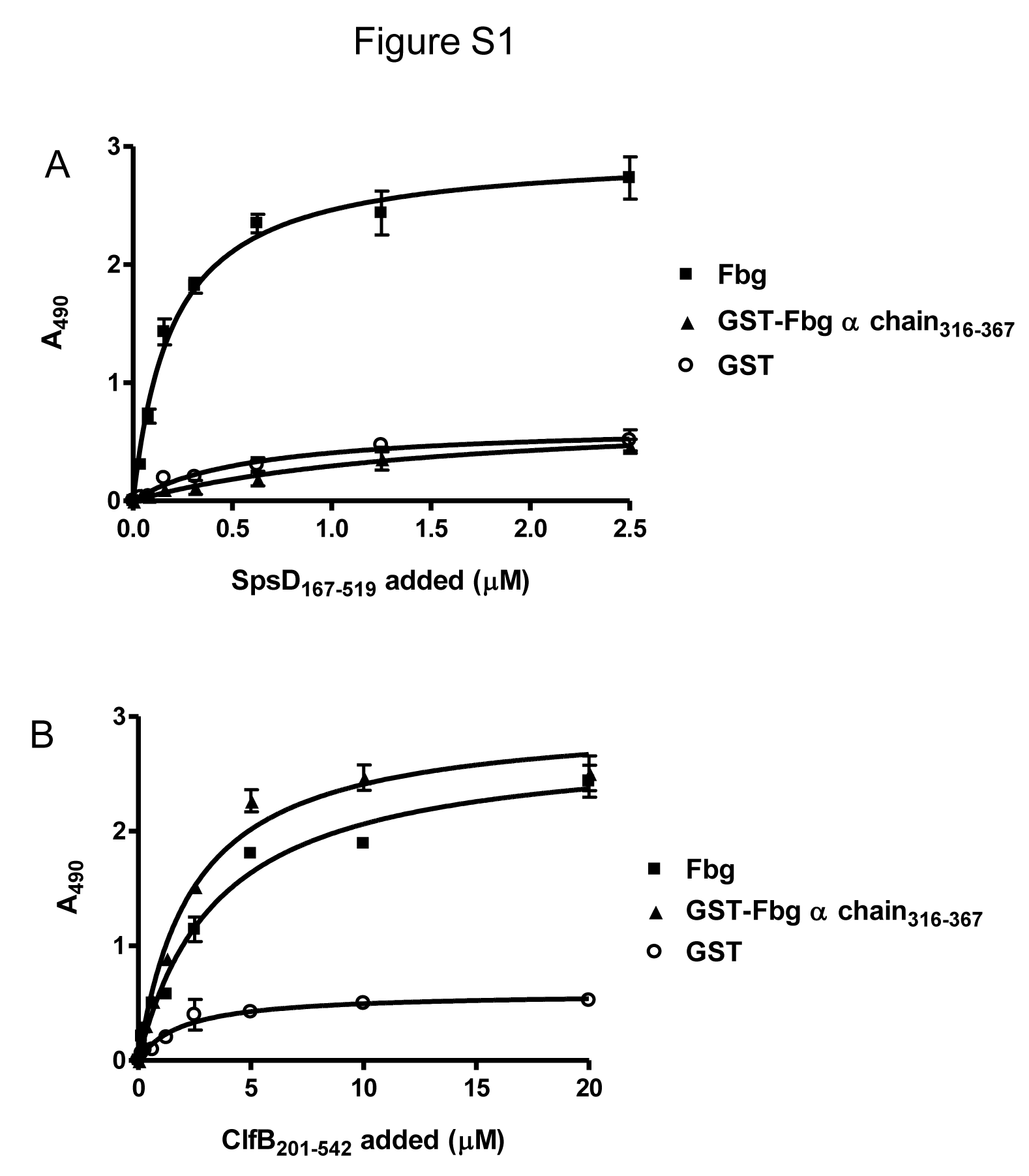

Supplement: Figure S1 — Binding of SpsD167–519 or ClfB201–542 to Fbg and Fbg α chain. Increasing concentrations of SpsD167–519 (A) or ClfB201–542 (B) were incubated in wells coated with 500 ng/well human Fbg, GST in fusion with the α chain peptide (aa 316–367) or GST alone. Bound ligand was detected by addition of mouse anti-SpsD37–519 IgG or anti-ClfB45–542 IgG followed by HRP-conjugated rabbit anti-mouse IgG. Results shown in the panels are the mean values of triplicate samples ± S.E. The experiments were repeated three times with similar results. (TIF) [file pone.0066901.s001.tif]

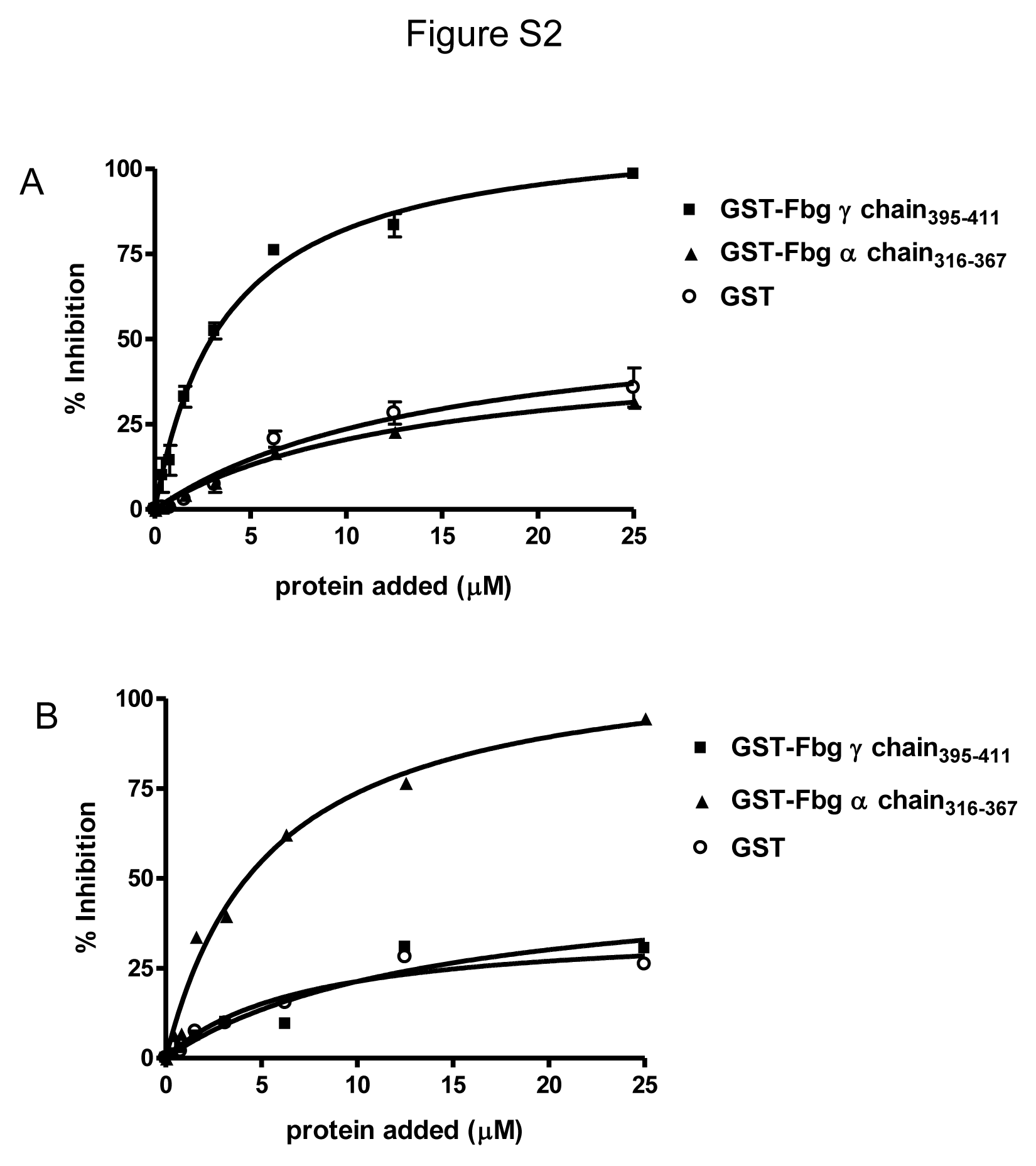

Supplement: Figure S2 — Effect of Fbg recombinant chains on the binding of SpsD167–519 or ClfB201–542 to surface-coated Fbg. 500 ng SpsD167–519 (A) or ClfB201–542 (B) mixed with increasing amounts of GST-tagged α chain, GST in fusion with the C-terminus of the γ chain, or GST alone were added to and incubated with microtiter wells coated with Fbg (500 ng/well). Bound ligand was detected by addition of mouse anti-SpsD37–519 IgG or anti-ClfB45–542 IgG followed by HRP-conjugated rabbit anti-mouse IgG. Results shown in the panels are the mean values of triplicate samples. Error bars show the standard deviation. The experiments were repeated three times with similar results. (TIF) [file pone.0066901.s002.tif]
